# The Use of Filtered Radiometers for Radiance Measurement

**DOI:** 10.6028/jres.116.018

**Published:** 2011-10-01

**Authors:** Albert C. Parr, B. Carol Johnson

**Affiliations:** Space Dynamics Laboratory 1695 N. Research Park Way Joint NIST/USURF Program in Optical Sensor Calibration, Logan, Utah 84341; Optical Technology Division, National Institute of Standards and Technology, Gaithersburg, MD 20899

**Keywords:** calibration coefficients, detector-based calibrations, filtered radiometer, mean wavelength, radiometry, spectral radiance

## Abstract

A methodology for using a calibrated filter radiometer to measure and monitor the spectral radiance of calibration sources is described. An example is presented using the NIST calibration sphere source that is used to support the NASA Earth Observing remote-sensing program.

## 1. Introduction

**Fig. 1:**
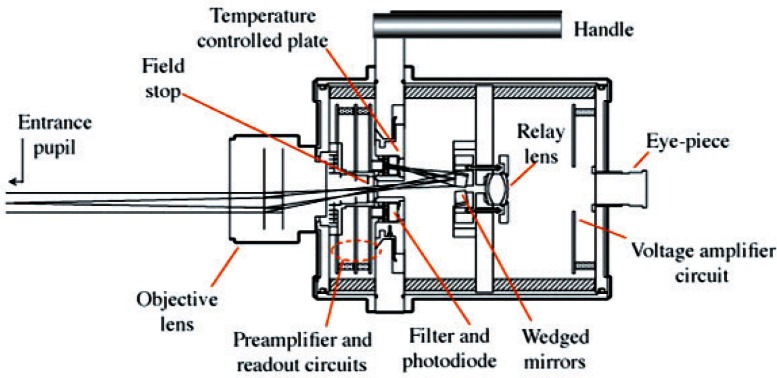
Cross sectional diagram of the VXR instrument showing the major optical elements.

The use of filtered radiometers in a wide range of radiometric activities has been made possible by the advent of stable, reliable, and inexpensive solid state detectors [1]. A filtered radiometer consists of an optical filter designed to transmit in a selected wavelength range and a photodetector, most commonly a silicon photodiode or other solid-state device, to detect the transmitted radiation. In some cases multiple detectors and filters are employed to give information about a spectral distribution. The Visible Transfer Radiometer (VXR) developed by the National Institute of Standards and Technology (NIST) to support the National Aeronautics and Space Administration’s (NASA’s) Earth Observing System (EOS) remote-sensing program is an example of such a device and is the subject of discussion for this paper [2]. The VXR is shown in Fig. 1. It consists of an objective lens that focuses the incident radiation onto a set of six filtered detectors. The radiation is reflected by a set of mirrors and the system features an optical alignment system for aligning with the source of the emitted radiance. The outputs of the detectors are amplified by an electronic system and an output voltage is provided that is proportional to the power of the incident radiation.

**Fig. 2:**
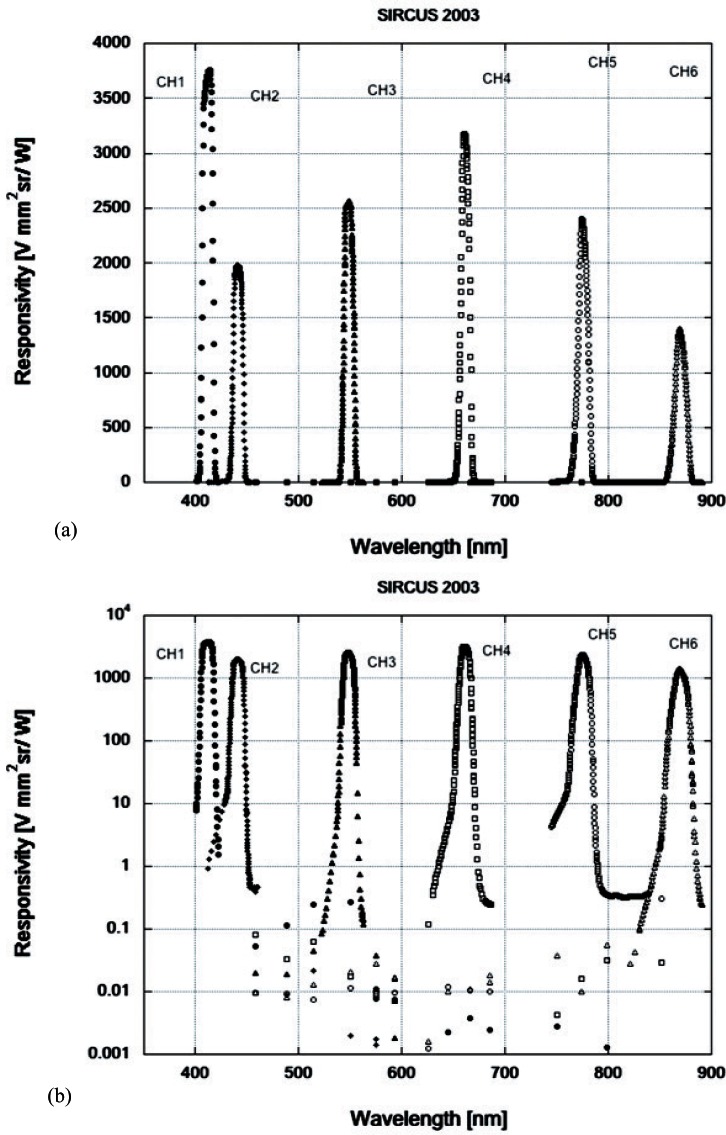
The responsivity of the 6 VXR channels as determined using SIRCUS. a) linear scale; b) logarithmic scale. The out-of-band response is 10^–5^ or less from the peak and hence negligible for the purposes of this paper.

To use the VXR as a radiance detector, it must be calibrated using a known source of spectral radiance. This is conveniently accomplished by the use of a facility at NIST developed for this purpose and called the facility for Spectral Irradiance and Radiance responsivity Calibrations using Uniform Sources (SIRCUS) [3]. The SIRCUS facility features a suite of tunable laser sources that provide radiation from 250 nm through a portion of the infrared region. The VXR operates between 400 nm and about 900 nm, a region entirely covered by the SIRCUS laser sources and transfer standard detectors. The VXR responsivity as measured on SIRCUS in 2003 is shown in Fig. 2a using a linear scale and in Fig. 2b using a logarithmic scale. The log plot shows the dynamic range of the SIRCUS calibration and its ability to provide the out-of-band response of the instrument. The six filters have center wavelengths of about 412 nm, 441 nm, 548 nm, 661 nm, 776 nm and 870 nm. The electronic control system is automated and the six voltages from the amplifiers are recorded by a computer system for analysis and subsequent use by the experimenters.

This paper is primarily intended to develop a set of parameters that can be used to measure the radiance of a black body type source at the VXR wavelengths if the radiometer is appropriately calibrated. In the case of the VXR, since it has six filtered detectors, the radiance of the source can be determined at six wavelengths and using interpolation methods, the spectral radiance of the source can be determined. This is not the central focus of the paper however and its details will be pursued in a later publication.

## 2. Methodology

The output signal for a particular channel can be related to the throughput of the channel, the magnitude of the incident spectral radiance, the detector spectral flux responsivity, and some geometric factors. Reference 2 gives an expression for the output signal *S* in volts of a filtered radiometer as,

(1) $$S=A\Omega G\int \tau (\lambda )R_{f}(\lambda )L_{\lambda }(\lambda )d\lambda ,$$

where *A*Ω is the throughput in square millimeter steradian, *G* is the amplifier gain in volts per ampere, τ(λ) is the spectral transmittance, *R*_f_(λ) is the spectral flux responsivity of the detector in ampere per watt, and *L*_λ_(λ) is the spectral radiance in watt per nanometer per square millimeter per steradian. Other combinations of the fundamental units can be used for specifying these parameters by using appropriate scale factors. The factor *R*_f_(λ) is measured at NIST (using a detector characterization facility) [4, 5] and should not be confused with the spectral radiance responsivity which is introduced below.

The throughput and transmission are properties of the optical system. The gain factor is a property of the built-in preamplifier and the voltage gain electronics which generates a voltage signal proportional to the flux at the silicon photodiode. The gain factor contains dimensionless ratios of resistances depending on the scale factor used in the measurement. The bandpass is defined by the filter transmittance and is on the order of 10 nm for the VXR channels. The integral over the wavelength sums the total power transiting the system and incident upon the photodetector within its range of sensitivity.

The SIRCUS calibration utilizes laser sources that are directed to an integrating sphere with an output aperture that is viewed by the VXR. In order to minimize uncertainty due to procedural differences, the calibration configuration is similar to that in which the VXR is utilized in the laboratory to monitor radiance from a calibration sphere. As the VXR is used, the geometric factors in Eq. (1) can be combined into an absolute radiance responsivity *R_i_*(λ) for the *i*th channel, given by,

(2) $$R_{i}(\lambda )=A_{i}\Omega_{i}G_{i}\tau_{i}(\lambda )R_{f,i}(\lambda ),$$

The factors on the right hand side of Eq. (2) are subscripted by *i* to indicate that all these quantities are potentially different for each channel of the VXR. Due to the built-in electronics, the signal output is a voltage and hence the responsivity to monochromatic flux is in the units of volts divided by radiance. The built-in voltage gain amplifier is operated with nominal gains of unity, 10, 100, or 1000, with the gain range correction factors determined in a separate characterization and the absolute spectral responsivity *R_i_*(λ) referenced to the unity gain ratio. Using this definition of the absolute radiance responsivity we can write a measurement equation for the output signal *S_i_* for each channel *i*, as

(3) $$S_{i}=\int R_{i}(\lambda )L_{\lambda ,s}(\lambda )d\lambda ,$$

where *R_i_*(λ) is the absolute radiance responsivity defined in Eq. (2) for the *i*th channel and unity gain and *L*_λ,s_(λ) is the spectral radiance of the test source.

To evaluate the integral expression, knowledge of *L*_λ,s_(λ) is required. However, by defining some parameters that define the integral in cases where we know the spectral radiance allows us to determine the radiance in other situations where certain criteria about the nature of the source are fulfilled.

The output *S_i_* from Eq. (3) is an integral over a distribution formed by the responsivity and the spectral radiance. It is then useful to calculate some mean parameters for this distribution which will allow a simple approximation of the integral. We first define the mean wavelength, λ_m,_*_i_*, of channel *i* as,

(4) $$\lambda_{m,i}=\frac{\int \lambda L_{\lambda ,s}(\lambda )R_{i}(\lambda )d\lambda }{\int L_{\lambda ,s}(\lambda )R_{i}(\lambda )d\lambda }.$$

The range of the integrals is over the contributing part of the distribution. It is important to be mindful of the possibility of long tails and secondary transmission windows in filters, which can make significant contributions to the integrals involved. Therefore, it is important the filter detector system be characterized over the range of the sensitivity of the detector and the range of the broadband incident spectra in order to avoid errors in the use of the VXR, or any multi-band filter radiometer, as an absolute radiometer. This problem is avoided in the VXR as the filters have excellent out-of-band suppression as indicated in Fig. 2b.

**Fig. 3:**
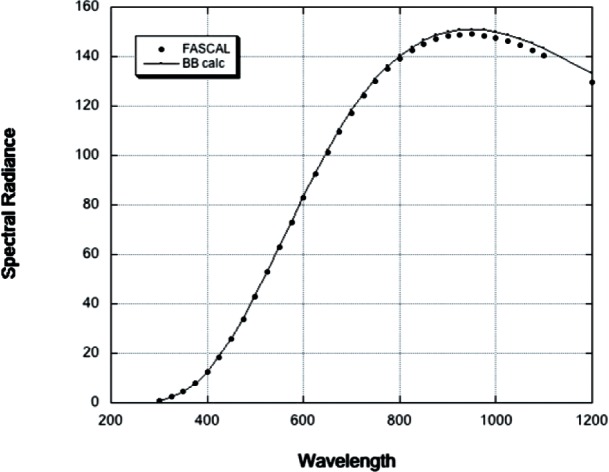
Spectral radiance from a black body at 3061 K (solid line) and the NPR integrating sphere source (2003 calibration, solid circles). The units on the vertical axis are μW/(nm sr cm^2^).

The VXR is primarily used to validate integrating sphere radiance sources that use quartz halogen lamps as the source of radiation. Figure 3 presents the spectral radiance of the NIST Portable Radiance source (NPR) as determined in 2003 by the NIST FASCAL facility and the scaled spectral radiance of a 3061 K black body (BB) which are plotted on the same axes [6, 7]. The BB curve in this plot is found from fitting Wien’s law to the NPR output data and using the temperature (3061 K) as determined by fitting the data. For this exercise only the wavelength region below 1000 nm was important since the silicon photodiode detectors are expected to cut off sharply above this wavelength, which makes it unnecessary to fit the NPR spectra over its entire range. The fitting using a simple model is adequate for our purposes and will allow us the use a BB spectrum in Eq. (4) to calculate the mean wavelength. We will later show that the uncertainties introduced by this are minimal when compared to using the actual FASCAL measured spectral radiance. It is important, when using a BB approximation for the actual spectral distribution of the source, to be mindful that the relative spectral distribution for the BB model is a function of the BB temperature, and this relative spectral distribution may not adequately describe the laboratory source over the entire spectral region of interest. It is also possible to have spectral features in the laboratory source that will not be present in the BB model, e.g. absorption in the sphere wall coating, atmospheric absorption, or spectral features in the quartz halogen lamps.

To simplify calculations, we introduce the concept of the effective width of the distribution in order to approximate the integral in Eq. (4) by a product of factors. We define the effective width for the channel *i* as Δλ*_i_*, where Δλ*_i_* is defined in the following equation:

(5) $$\Delta \lambda_{i}L_{\lambda }(\lambda_{m,i})R_{i}(\lambda_{m,i})=\int L_{\lambda }(\lambda )R_{i}(\lambda )d\lambda ,$$

Solving for the effective width we obtain

(6) $$\Delta \lambda_{i}=\frac{\int L_{\lambda }(\lambda )R_{i}(\lambda )d\lambda }{L_{\lambda }(\lambda_{m,i})R_{i}(\lambda_{m,i})}.$$

Note that Eq. (5) is exact; other choices for definition of the mean wavelength (see Eq. (4)) would result in different numerical values for the quantities on the left hand side of Eq. (5), but the product is constant. Using readily available computational software such as Mathematica,^1^ the integrals can be easily performed by numerical techniques [8]. This parameterization expresses the value of the integral in Eq. (5) by the value of the distribution, *L*_λ_(λ) *R_i_*(λ), at its mean wavelength times the effective width. Note that both the effective width and the mean wavelength defined above are formed by ratios and hence are only sensitive to the shape of the spectral radiance and are not the absolute values.

As an alternative to using a BB spectrum for the evaluation it is possible to use the spectral radiance data that NIST supplied with the calibration of the sphere. The calculation is done using an interpolation routine for the spectral radiance calibration data and performing the numerical integration using these interpolated results. This differs slightly from using an analytical function such as the Planck function in the integration routine but does not appreciably affect the results for λ_m,_*_i_* or Δ_λ_*_i_*, and hence either can be used. This is discussed later in more detail.

The utility of the parameterization is evident from combining Eqs. (3) and (5), where solving for the spectral radiance, we get

(7a) $$L_{\lambda ,s}(\lambda_{m,i})=\frac{S_{i}}{\Delta \lambda_{i}\;R_{i}(\lambda_{m,i})},$$

This relationship allows us to express the spectral radiance of a source directly in terms of fixed parameters of the radiometer and the measured signal. That is, the denominator on the right of Eq. (7a) can be considered a calibration constant *C_i_*, of the radiometer, and we can write

(7b) $$C_{i}=\Delta \lambda_{i}\;R_{i}(\lambda_{m,i}).$$

This relationship shows that once the mean wavelength and effective width are determined, that the VXR can be used to ascertain the spectral radiance of any source at mean wavelengths, λ_m,_*_i_* assuming the source has a spectral distribution similar to that of the one used to calculate these values. This makes the VXR a potentially useful device to accurately track calibration spheres used in field calibration of radiometers in many remote-sensing programs.

NIST has used the VXR and an earlier, similar, instrument, the SeaWiFS Transfer Radiometer (SXR) [9, 10], to validate sphere sources for various programs in support of Earth observation [11, 12]. In addition, the SXR or the VXR have been deployed to the calibration facility of the Marine Optical Buoy (MOBY) program starting in 1996 to validate the spectral radiance values of the MOBY calibration sources [13] by comparison of actual measured signals to those expected from Eq. (3). The validation activity requires that the participant supply the values *L*_s_(λ) for the sphere source under consideration to NIST prior to the VXR measurements of that source for use in Eq. (3). These activities began prior to SIRCUS and its ability to provide absolute radiance responsivities, so measurements of the relative spectral radiance responsivity and NIST reference spheres were used to derive the calibration coefficients [2, 9].

Reference [9] described the use of the SXR as an absolute radiometer based on these source-based calibration coefficients and presented an uncertainty budget for that approach. In this work, we present a detector-based method of producing calibrating coefficients that differs from that described in Ref. [2] by the use of a BB distribution to account for the spectral distribution of the source under consideration. The suggested method is a way to determine absolute spectral radiance values, Eq. (7) that takes full advantage of the SIRCUS uncertainties while also including the effects of the source’s relative spectral distribution. Also, the long-term behavior of the source can be assessed independently from the supplied values for *L*_λ,s_(λ). This would work even if the sphere had lamp changes as long as the spectral shape did not change significantly. In using a VXR-type device as a monitor of source stability it is important to validate the stability of the VXR’s calibration and make any adjustments as required to the calibration constants. For example, if the responsivity of a channel drifted due to filter or detector changes, the responsivity could be remeasured and new calibration constants determined. The parameterization suggested here might offer a simple and robust approach to the estimation of the temporal behavior of the VXR spectral responsivity, e.g., by fitting the mean wavelength, effective width, and calibration constant determined with the SIRCUS measurements to analytical functions that best represent device performance.

As an example of the usefulness of the suggested approximation, the values of the mean wavelength, effective width and calibration constant were calculated for both distributions shown in Fig. 3. The results are shown in Table 1. The calculated results for the NPR sphere spectral distribution are shown in the column labeled NPR and that of a BB at 3061 K in the column labeled black body. Inspection of the table shows that the differences between the NPR and the BB calculations are very small. The differences in the mean wavelengths and the effective widths are less than the uncertainties associated with the FASCAL wavelength scale of the source under measurement and hence in this case do not contribute any significant uncertainty. The differences in the calibration constants are less than 0.2 % in all cases and hence in this particular example one could expect agreement between the results to be close regardless of which spectral distribution used for the calculation of the relevant quantities. As a further test the calibration constants were calculated using a FASCAL calibration of the NPR sphere performed in June 2009 and the same SIRCUS 2003 data. The differences between the values calculated using the 2003 and 2009 calibration of the NPR differed by less than 0.1 % for all the calibration constants except *C*_6_, the value for the 869.7 nm channel, which was 0.11 %.

Therefore, in a case like this where the out-of-band spectral responsivity in the filtered detector is orders of magnitude less than the in-band responsivity, the sphere output is close to a BB distribution, and the correct BB temperature is used, other sources of uncertainty will dominate the uncertainty budget, such as uncertainties from size-of-source effects, uniformity in the sphere’s spectral radiance at the exit aperture, measurement repeatability, and so forth. If the relative spectral shape of the sphere’s spectral radiance stays the same over time, the VXR is stable over time, and the measurement repeatability is sufficient, then the use of values calculated initially could be used to monitor the long-term behavior of the sphere output with very good accuracy. If calibration of a sphere indicates that its spectral shape has varied significantly, then new VXR calibration constants can be calculated using the new spectral radiance spectrum of the sphere or of an equivalent BB.

The monitoring measurements will give one value of the spectral radiance at the six wavelengths given by λ_m,_*_i_* for each channel of the VXR. These six points can be used to calculate the shape of the rest of the spectral distribution by fitting the six points to a modified BB spectra using the Planck or Wien law as desired, thus giving the whole spectral radiance output of the sphere. The six wavelengths of the spectral response are the six mean wavelengths defined above.

While the sensitivity of the calculation of the values of *C_i_* are relatively insensitive to the NPR calibration or an equivalent blackbody, they are sensitive to the VXR calibration. This would be expected as the responsivity is the dominant varying factor in the integrals. The spectral radiance change is gradual over the wavelength interval where there is significant filter transmittance, an issue to which we shall return.

## 3. Discussion of Usage

The quantities *C_i_* are useful for several purposes. First, from VXR measurements, they allow a direct computation of the spectral radiance of the integrating sphere source for which they have been previously calculated, see Eq. (7a). The VXR could be employed on a routine basis for checking if the sphere calibration is drifting or if problems have developed. Secondly, it is a useful metric on the stability of the VXR in that it is the quantity that defines the relationship for using the VXR to determine spectral radiance. If the quantity *C_i_* changes by some amount as the result of a calibration, one then has a direct measure of how much the VXR measurement of a particular sphere source is going to change. Information on such a change offers a mechanism for tracking changes in a historical record.

As an example of the use of the first technique, we will use a calibration of the VXR in 2003 by SIRCUS and the spectral radiance of the NPR sphere calibrated by FASCAL in 2003. The calibration of the sphere by FASCAL and the calibration of the VXR and its use in measuring the NPR were done in about the same time. While there may be drifts of both the NPR and VXR over the time the data was taken, the data will provide useful means to explore the methodology suggested here. It also shows how to use the methodology to look at historical calibration records where there are VXR measurements of an integrating sphere in between the NIST calibration of the sphere. Table 2 shows the results of this computational exercise. The column marked *L*_VXR_(λ_m,_*_i_*) is the radiance of the NPR calculated using the VXR measurements and the SIRCUS calibration with the methodology suggested here– Eq. (7a). The column labeled *L*_s_(λ_m,_*_i_*) is the spectral radiance determined on FASCAL for the NPR interpolated to the mean VXR wavelength–Eq. (4). The percentage differences of these results, which correspond to the measured vs. expected spectral radiances, are shown in the last column.

In the blue region, where the spectral radiance is lowest and the calibration most uncertain, the discrepancy is largest. In the visible spectral region, the agreement between the methods is quite good. However at the extremities, (the 412 nm and the 869 nm channels), the differences are larger than expected, as suggested by the FASCAL scale uncertainties and the estimated biases from using the method outlined here. The uncertainties of the FASCAL calibration are between 0.6 % and 1.3 % (*k* = 2) at 400 nm and in the visible and near infrared they are about 0.5 % to 0.8 % (*k* = 2). Additional components of uncertainty include the uniformity of the radiance in the exit aperture (FASCAL and the VXR differ in the target area), the repeatability of VXR-NPR measurement system, and the uncertainty in the SIRCUS *R_i_*(λ) values, which are about 0.2 % (*k* = 2). These differences will warrant further study as we pursue implementation of the suggested procedures. The example shown in Table 2 is intended to show the ease of use of the method—the values for the spectral radiance determined by the VXR are simply the result of dividing the calibration factors into the voltage output by the VXR electronics. These six values of the spectral radiance can then be used to get the spectral radiance over the range covered by fitting the six points to a blackbody type function or and an experimentally determined numerical model for the spectral radiance of the sphere [7].

It would help the effort tremendously if the VXR could be checked in the laboratory using a known and stable standard validation source that could be tied to the NIST primary optical watt radiometer (POWR) [14] and thereby track the VXR performance to detector standards. The absolute detector-based spectral radiance source under investigation at NIST may be able to serve such a purpose [15]. It would be expedient if this chain of calibration, or something equivalent, could be developed without necessitating a complete recharacterization by the SIRCUS team as this is time-consuming and is difficult to schedule because of the high demands on the SIRCUS facility for a number of fundamental calibrations.

## 4. Uncertainties

In order to obtain an estimate of the inherent uncertainties in the results for this methodology for determining the spectral radiance of an integrating sphere source, we undertook a number of computer simulations under various conditions. The mean wavelength using the FASCAL calibration data for the NPR compared to the BB model only differed by a few hundredths of a nanometer. A difference of this amount results in little change in expected spectral radiance of a sphere source. A series of calculations using the 2003 SIRCUS calibration was also performed. The calculations involved the determination of the calibration coefficient, *C_i_*, for all the channels and for BB temperatures from 2200 K to 3200 K. This range includes likely effective temperatures of sources that the VXR could measure. Also, the mean wavelengths were calculated for all these situations. The results are shown in Tables 3 and 4.

The largest differences are in *C*_1_, which changes by about 1.5 % between 2200 K and 3200 K. The other calibration constants vary significantly less. This is expected since the short wavelength part of the BB distribution is changing the most for this temperature interval. The calibration constants for channels three through six change by less than 0.1 % over this temperature range. The data in Table 3 are sufficient to calculate the temperature coefficient of change for the calibration constants if it is needed for some correction due to varying source conditions. It is a simple matter to make a table of numbers of the effective source temperature and calibration constant for a given channel and perform a polynomial fit to the results at the six BB temperatures to generate the calibration coefficient for any temperature in the range of 2200 K to 3200 K. We found a quadratic equation was all that was necessary to achieve good results. Note that the spectral radiance emitted by a BB will change substantially over this temperature range, because of the strong dependence upon temperature by the Planck law.

**Fig. 4:**
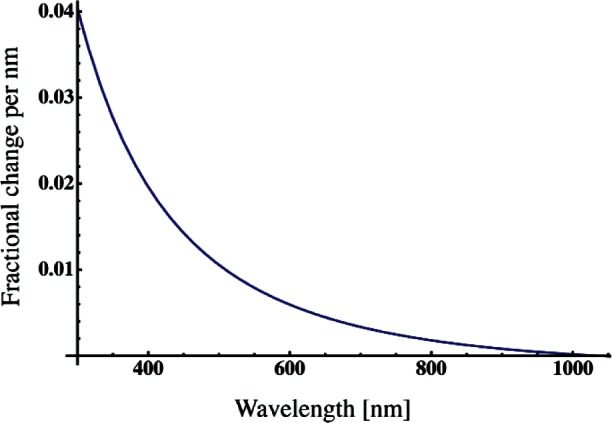
The fractional change in spectral radiance per nanometer for a black body at 2800 K.

Likewise the greatest sensitivity to BB temperature is for λ_m,1_, and it changes by 0.65 nm for the entire temperature range. The BB spectral radiance for this temperature range, as well as typical integrating sphere spectral radiance values, varies with wavelength more rapidly in the blue than at longer wavelengths. Therefore an error in λ_m,_*_i_* at 412 nm will result in a larger bias than an error at 870 nm. To estimate this effect, the fractional change in the BB spectral radiance at 2800 K as a function of wavelength was calculated (see Fig. 4). When multiplied by the wavelength change, this gives the fractional change in spectral radiance caused by a given wavelength shift. At 400 nm, the fractional change is 0.02 and the wavelength shift caused by a 200 K temperature change is about 0.1 nm (Table 4). A change in 200 K would then introduce a bias of about 0.2 %. The bias at the other channels is less, as can be seen by the decrease of the fractional change with wavelength. A conclusion is that small changes of a few hundred Kelvin have a small to negligible effect upon the VXR’s ability to accurately determine the spectral radiance of a source as described in this paper.

A better feel for the intrinsic uncertainties attained when using this method will emerge after some use but we expect that, if a good model for the spectral radiance is used for the calculation of the mean wavelength, effective width and calibration constant, then the VXR can be used to determine (and hence monitor) the absolute spectral radiance of an integrating sphere without adding significant uncertainty to the overall VXR-sphere uncertainty budget. This overall budget must include uncertainty components associated with channel-dependent temporal changes in the VXR spectral radiance responsivity as well as drift in the spectral radiance of the integrating sphere.

## 5. Conclusions

A self-consistent methodology has been developed to use a filtered detector to determine the spectral radiance and the associated temporal stability of lamp-illuminated integrating sphere sources. In particular the VXR radiometer used by NIST has been analyzed and demonstrated to be capable of useful and accurate measurement of the NPR sphere. Since the method is dependent only on the shape of the spectral distribution, not its magnitude, in those cases where a sphere sources has the ability to use various numbers of lamps to vary signal levels, the method should work well at reduced signal levels. This has not been checked at this point and will be the subject of a future study. This same technique could be used to monitor other spectral radiance sources where the spectral distribution is roughly Planckian as is the case when tungsten halogen lamps are employed as the primary source. The computations involved are minimal and rely upon commercial software with programs written that utilize built-in functions and capabilities of the software such as linear regression, interpolation and numerical integration. If this procedure were embedded in routine analysis of the long-term validation exercises, the performance of an integrating sphere source that serve as the facility’s radiance reference standards could be assessed without *a priori* knowledge of the source’s spectral radiances as long as the general shape of the spectral output was similar to that with which the parameters were derived. In the MOBY case, a secondary laboratory initially provided the spectral radiance calibrations for the MOBY reference standards, and these values had uncertainties that are greater than the uncertainties furnished currently by the NIST calibrations of these artifacts. A planned area of research is to determine the uncertainties associated with using the VXR time series and the methodology presented here in order to reduce the uncertainties of the MOBY spectral radiance responsivity during the interval when these values were established using the secondary laboratory calibrations. For this and any related effort, it will be critical to maintain a record of the VXR calibration and any temporal changes in its responsivity. If the calibration of the VXR’s individual channels have changed, it will be necessary to recalculate the calibration coefficients in order to ensure accuracy.

The use of the VXR or equivalent filter detectors in the manner suggested here offers a direct way of comparing the spectral radiance scales derived from detector based calibrations like SIRCUS and the traditional lamp radiance sources used in FASCAL. It can be another tool to maintain calibration accuracy of integrating sphere sources deployed in laboratories and for which routine calibrations at NIST or other standards laboratory may not be feasible.

## Figures and Tables

**Table 1:** Comparison of the calculated values of a) the mean wavelength, λ_m,_*_i_*, b) the effective width, λ*_i_*, and c) the calibration constants, *C_i_*, for the spectral distribution from the NPR integrating sphere source with a 2003 FASCAL calibration and a black body distribution at 3061 K, both using 2003 SIRCUS calibration data for the VXR

a)			
Channel *i*	Source		Comparison

	NPR λ_m,_*_i_* (nm)	Black body λ_m,_*_i_* (nm)	(NPR-BB)/NPR (percent)
1	411.683	411.647	0.009
2	441.010	440.993	0.004
3	548.225	548.216	0.002
4	661.078	661.072	0.0001
5	775.099	775.098	0.0001
6	869.522	869.522	0.0

b)			
Channel *i*	Source		Comparison

	NPR Δλ*_i_* (nm)	Black body Δλ*_i_* (nm)	(NPR-BB)/NPR (percent)
1	10.715	10.737	−0.205
2	10.311	10.319	−0.078
3	10.247	10.246	+0.010
4	9.323	9.323	+0.0
5	11.103	11.106	−0.027
6	13.120	13.120	+0.0

c)			
Channel *i*	Source		Comparison

	NPR C*_i_* (V cm^2^ sr nm/W)	Black body Cλ*_i_* (V cm^2^ sr nm/W)	(NPR-BB)/NPR (percent)
1	39979	40059	−0.200
2	20443	20461	−0.088
3	26198	26196	+0.008
4	29437	29437	+0.0
5	26340	26346	−0.023
6	18050	18049	+0.006

**Table 2:** Sample results from using the VXR to measure the spectral radiance of the NPR integrating sphere source. The mean wavelength, measured voltage, and 2003 SIRCUS calibration coefficients are given along with the measured and expected spectral radiances and the differences in percent: 100 % [*L*_λ,VXR_(λ_m,_*_i_*) − *L*_λ_,_s_(λ_m,_*_i_*)] / *L*_λ,s_(λ_m,_*_i_*)

Channel *i*	λ_m,_*_i_* (nm)	*S_i_* (V)	*C_i_* (V cm^2^ sr nm/W)	*L*_λ,VXR_(λ_m,_*_i_*) μW/(cm^2^ sr nm)	*L*_λ,s_(λ_m,_*_i_*) μW/(cm^2^ sr nm)	Diff. (%)
1	411.68	0.4419	39979	11.05	11.27	−1.95
2	441.01	0.3710	20443	18.15	18.25	−0.55
3	548.23	1.4822	26197	56.57	56.57	+0.0
4	661.08	3.0427	29437	103.36	103.23	+0.13
5	775.10	3.6562	26340	138.81	137.02	+1.31
6	869.52	2.7174	18050	150.55	149.01	+1.03

**Table 3:** The calculated calibration constants *C_i_* for the SIRCUS 2003 results as a function of BB temperature for the VXR channels, see Eq. (7b)

	*C_i_* (V cm^2^ sr nm / W)					

Channel	1	2	3	4	5	6
	
Temperature (K)						
2200	39442	20493	26175	29442	26510	18050
2400	39710	20511	26182	29446	26515	18051
2600	39862	20524	26187	29449	26516	18052
2800	39956	20534	26191	29452	26520	18053
3000	40017	20547	26193	29454	26522	18054
3200	40060	20548	26192	29456	26523	18054

**Table 4:** The calculated mean wavelength λ_m,_*_i_* for the SIRCUS 2003 results as a function of BB temperature for the VXR channels, see Eq. (4)

	λ_m,_*_i_* (nm)					

Channel	1	2	3	4	5	6
	
Temperature (K)						
2200	412.51	441.07	548.29	661.14	774.98	869.55
2400	412.26	441.03	548.27	661.12	774.95	869.53
2600	412.11	441.00	548.25	661.10	774.93	869.51
2800	412.00	440.98	548.24	661.09	774.90	869.50
3000	411.92	440.95	548.22	661.08	774.88	869.48
3200	411.86	440.93	548.21	661.07	774.86	869.47

## Footnotes

1 Certain commercial equipment, instruments, or materials are identified in this paper to foster understanding. Such identification does not imply recommendation or endorsement by the National Institute of Standards and Technology, nor does it imply that the materials or equipment identified are necessarily the best available for the purpose.
